# Ultrasound of the hand is sufficient to detect subclinical inflammation in rheumatoid arthritis remission: a post hoc longitudinal study

**DOI:** 10.1186/s13075-017-1428-4

**Published:** 2017-10-04

**Authors:** Hilde Berner Hammer, Tore K. Kvien, Lene Terslev

**Affiliations:** 1 Department of Rheumatology, Diakonhjemmet Hospital, Box 23 Vinderen, 0319 Oslo, Norway; 2 Centre for Rheumatology and Spinal Diseases, Copenhagen University Hospital Rigshospitalet, Copenhagen, Denmark

**Keywords:** Rheumatoid arthritis, Ultrasound, Inflammation, Synovium, Hand, Biologic therapies

## Abstract

**Background:**

Ultrasound (US) is a sensitive method for detecting joint/tendon inflammation in patients with rheumatoid arthritis (RA). Subclinical inflammation is often found in patients with RA in composite score remission. The purpose of the present study was to explore whether US of only the hands is sufficient to identify subclinical inflammation in patients with established RA in clinical remission.

**Methods:**

A total of 209 patients with established RA (81% women, mean [SD] age 53.3 (13.2) years, disease duration 10.0 [8.8] years) were examined when initiating biologic disease-modifying anti-rheumatic drugs (bDMARDs) and after 6 months (184 patients) and 12 months (152 patients) of follow-up. They were assessed by US (greyscale [GS] and power Doppler [PD] of 36 joints and 4 tendons, scored 0–3) as well as clinical and laboratory examinations, and different disease activity composite scores were calculated. The presence of US synovitis (GS score ≥ 2, PD score ≥ 1 [PD1] and score ≥ 2 [PD2]) in composite score remission was explored.

**Results:**

Remission at 6 and 12 months was achieved in 74 and 59 patients, respectively, for Disease Activity Score based on 28 joints (DAS28); in 37 and 38 patients, respectively, for Clinical Disease Activity Index; in 42 and 42 patients, respectively, for Simplified Disease Activity Index; and in 38 and 35 patients, respectively, for Boolean remission. The percentages of patients in DAS28 remission at 6 months with synovitis in hands/other regions were 73.0%/64.9% for GS, 64.9%/41.9% for PD1 and 32.4%/20.3% for PD2; at 12 months, the corresponding percentages were 61.0%/64.4% for GS, 62.7%/39.0% for PD1 and 44.1%/15.3% for PD2, respectively. PD activity was more often present in the hands (*p* < 0.001). In patients in various composite scores of remission, US only of the hands identified ≥ 90% of the patients having PD activity in any of the assessed joints/tendons.

**Conclusions:**

A high percentage of patients had US synovitis despite being in clinical remission. US examination performed only of the hands captured ≥ 90% of patients with subclinical inflammation and could be feasible for assessing bDMARD-treated patients with RA in remission.

**Trial registration:**

Australian New Zealand Clinical Trials Registry, ACTRN12610000284066. Registered on 8 April 2010.

## Background

With the treat-to-target strategy (T2T), the updated treatment recommendations for rheumatoid arthritis (RA) in 2016 are aimed at remission within 3–6 months, though low disease activity may be an acceptable target in patients with RA with long-standing disease [1]. The current treatment strategy for patients with RA includes tight control with monotherapy or combination therapy using disease-modifying anti-rheumatic drugs (DMARDs). The aim of this strategy is to obtain rapid disease control, thereby preventing pain and joint destruction. Several clinical definitions of remission are proposed, mainly using composite scores where Boolean remission is the strictest [2–5].

Ultrasound (US) has been used as an instrument for monitoring disease activity in RA, where a synovial hypertrophy score ≥ 2 by greyscale (GS) and a power Doppler (PD) score ≥ 1 may be a sign of inflammatory activity. Recent studies have indicated that the presence of Doppler activity with a score of 1 may be seen in normal joints, suggesting a higher PD cut-off of ≥ 2 as a sign of pathology, though a certain cut-off between normality and pathology still needs to be determined [6–9].

Several reduced US joint sets have been proposed for optimising clinical utility, ranging from US assessment of 6- to 12-joint counts either unilaterally or bilaterally, all including hands and feet with or without tendon assessment [10–12]. The reduced scores have all been developed to retain as much information as possible from the more elaborate joint counts that may include up to 78 joints [10–12].

Though recent studies have shown that the added value of US assessments in a T2T strategy may be limited in very early RA if tight clinical control is applied [13, 14], several studies have shown that Doppler activity in patients in composite score remission predicts flare and radiographic progression, both at the patient and at the joint level [15–17]. It has also been found that, despite composite score remission, US joint inflammation is still frequent [18]. Hence, the most optimal situation is when patients are both in composite score and PD score remission, because this is found to cause a very low degree of radiographic progression [13].

A comprehensive US assessment is time-consuming, and clinical as well as US experience has shown that the hands are frequently involved in patients with established RA. Some of the proposed reduced joint sets have included tendons [12, 19], and the presence of tenosynovitis has been suggested to be related to flare in patients with RA in remission [20] and may thus be important in US assessments [21, 22].

The objective of the present study was to explore, in a post hoc analysis, the presence of GS and PD pathology in a high number of joints/tendons and to assess the frequency of US pathology in hands compared with other joints/tendons in patients with established RA in remission according to different composite scores. An additional objective was to explore whether PD assessment of the hands only is sensitive enough to detect subclinical inflammatory activity in patients with RA in composite score remission.

## Methods

The present cohort has previously been explored for the development of a reduced score set of joints and tendons for RA monitoring [12, 19]. Patients with RA fulfilling the American College of Rheumatology (ACR) 1987 criteria [23] were included in the study when initiating or changing biologic disease-modifying anti-rheumatic drug (bDMARD) treatment in the period from January 2010 to June 2013 (Australian New Zealand Clinical Trials Registry, ACTRN12610000284066).

### Clinical examinations

The patients were assessed at baseline and after 6 and 12 months by two trained study nurses with long experience in evaluation of tender and swollen joints, and the study nurses were blinded to the US findings. Patient and assessor global visual analogue scale (VAS) scores, as well as laboratory examinations (erythrocyte sedimentation rate [ESR] and C-reactive protein [CRP]), were assessed. The Disease Activity Score based on 28 joint counts with erythrocyte sedimentation rate (DAS28[ESR]) [2], Clinical Disease Activity Index (CDAI) [3] and Simplified Disease Activity Index (SDAI) [4] scores were calculated.

### US examinations

US examinations (blinded for the results of the clinical assessments) were performed as described previously [19]. In short, the same experienced sonographer (HBH) performed the US assessments throughout the study using a Siemens Antares Excellence version (Siemens Medical Solutions, Malvern, PA, USA) equipped with a 5- to 13-MHz linear probe, with 11.4 MHz used for GS assessments. PD settings were optimised for inflammatory flow with pulse repetition frequency 391 Hz, Doppler frequency 7.3 MHz and gain just below the level of noise [24]. The same US machine with the same settings for all joints and tendons was used throughout the study, with no software upgrades. US was performed on 36 joints and 4 tendons for signs of synovitis and tenosynovitis, including bilateral evaluation of the hands (metacarpophalangeal [MCP] joint 1–5 and proximal interphalangeal [PIP] joints 2 and 3, wrist [scoring radio-carpal, mid-carpal and radio-ulnar joints separately] and the extensor carpi ulnaris [ECU] tendon) as well as of other regions (elbow, knee, talocrural and metatarsophalangeal [MTP] joints 1–5, and the tibialis posterior [TP] tendon). Each joint/tendon was scored semi-quantitatively on a 0–3 scale for GS and PD as described previously [12, 19] and according to the US atlas by Hammer et al. [25] using the Outcome Measures in Rheumatology definitions for synovitis and tenosynovitis [26, 27]. The sonographer in the present study (HBH) had previously demonstrated a high intra-observer reliability for scoring of joints and tendons [19, 25].

GS synovial hypertrophy score ≥ 2 was defined as pathology, and for PD activity, two scores were explored: PD ≥ 1 and PD ≥ 2. The study was approved by the Regional Committee for Medical and Health Research Ethics South East (REK), and the patients gave written consent according to the Declaration of Helsinki (REK number 2009/1254).

### Statistics

Patients with at least one joint/tendon with GS score ≥ 2 or PD scores ≥ 1 or ≥ 2 in individual joints/tendons or groups of joints/tendons at baseline and at 6 and 12 months were identified. To explore the value of examining PD only of the hands (i.e., wrist, MCP joints 1–5, PIP joints 2–3, ECU tendon bilaterally), patients were identified with PD pathology detected in at least one joint/tendon in the hands and/or in at least one joint/tendon in the other sites. The percentages of patients with PD scores ≥ 1 or ≥ 2 in the hands and/or other sites were calculated for the whole cohort as well as for those in composite score remission. Remission criteria were used for DAS28 (<2.6), CDAI (≤2.8), SDAI (≤3.3) and ACR/European League Against Rheumatism Boolean remission (patient’s global VAS 0–10, tender and swollen joint counts and CRP [mg/dl] all ≤ 1) [2–5]. The Mann-Whitney *U* test was used to explore differences in frequencies of joint/tendon involvement. Missing data during follow-up (<5% missing values) were handled by use of the last observation carried forward. All tests for significance were two-sided, and *p* < 0.05 was considered significant.

## Results

### Patient characteristics and treatment

A total of 209 patients (81% women, mean [SD] age 53.3 [13.2] years, disease duration 10.0 [8.8] years, 79.2% anti-cyclic citrullinated peptide-positive and 68.6% positive for rheumatoid factor) were included [19]; 184 (87.1%) completed 6 months of follow-up; and 152 (72.7%) completed 12 months of follow-up. At baseline, the mean (SD) values of the composite scores were DAS28 4.55 (1.45), SDAI 21.2 (12.5) and CDAI 20.0 (11.8), whereas the sum US scores were 30.0 (18.9) for GS and 14.2 (13.6) for PD.

The patients initiated (and continued throughout the study) treatment with one of the following as their first (44.6%), second (30.1%), third (17.1%) or fourth to seventh (8.3%) bDMARD: etanercept (34.9%), rituximab (20.6%), certolizumab (11.0%), infliximab (10.0%), tocilizumab (8.6%), adalimumab (6.7%), golimumab (5.3%) and abatacept (2.9%). In addition, 83.6% were on synthetic DMARDs (90.6% of them used methotrexate), and 55% were on prednisolone with a mean (SD) dose of 8.0 (5.3) mg.

### Presence of US pathology in the whole cohort

Most of the US pathologies in the cohort were found in the joints of the hands and in MTP joints at both follow-up visits. Table 1 shows the percentages of patients with GS scores ≥ 2 as well as PD scores ≥ 1 or ≥ 2 in different joints and in groups of joints and tendons at baseline and at 6 and 12 months. The tendons were involved less frequently than the small joints but more often than the large joints. At baseline, there were significantly more patients with PD activity in at least one joint/tendon in the hands than in all the other joints/tendons (*p* < 0.001). Focussing only on the hands, we observed significantly more patients with PD activity in MCP joints and wrist joints than in the ECU tendon (*p* < 0.001), but we found no statistically significant difference between the presence of PD in MCP and wrist joints at any of the follow-up assessments.

**Table 1:** Patients with greyscale or power Doppler pathology in different joint/tendon regions at baseline and after 6 and 12 months

	Baseline (*n* = 209)			6 months (*n* = 184)			12 months (*n* = 152)		
	GS ≥ 2	PD ≥ 1	PD ≥ 2	GS ≥ 2	PD ≥ 1	PD ≥ 2	GS ≥ 2	PD ≥ 1	PD ≥ 2
Wrists (RC, MC, RU)	62.0	69.8	37.6	45.8	56.5	26.0	43.9	53.5	18.1
ECU	29.8	31.3	20.2	16.5	20.9	11.5	16.4	15.1	10.7
MCP 1–5	67.9	65.1	54.5	63.2	48.4	29.1	49.7	45.9	31.4
PIP 2–3	46.6	32.4	26.5	29.7	18.1	13.7	31.4	17.6	11.9
Elbow	20.2	19.7	12.0	15.5	11.0	3.9	13.8	9.4	5.0
Knee	18.1	9.8	2.9	6.2	1.7	1.1	5.1	3.2	0.0
Ankle	10.5	3.8	1.0	6.0	0.5	0.0	3.8	1.9	0.0
TP	27.4	32.2	21.2	18.7	20.9	13.2	10.1	11.9	5.7
MTP 1–5	78.5	56.5	38.0	65.1	37.8	16.4	61.7	28.9	12.1

*Abbreviations: RC* Radio-carpal joint, *MC* Mid-carpal joint, *RU* Radio-ulnar joint, *ECU* Extensor carpi ulnaris tendon, *MCP* Metacarpophalangeal joint, *PIP* Proximal interphalangeal joint, *TP* Tibialis posterior tendon, *MTP* Metatarsophalangeal joint

In the total cohort, GS scores ≥ 2 only in joints other than the hands were found in 9.9% of the patients at 6 months and in 12.6% at 12 months. At the joint level, GS scores ≥ 2 only in joints other than the hands were explained by MTP joint involvement in 15 of 18 patients at 6 months and in 19 of 20 patients at 12 months.

Table 2 illustrates the percentages of patients in the cohort at 6 and 12 months having PD activity in at least on joint/tendon only in the hands versus PD in at least one joint/tendon in any of the other areas, showing that not more than 4-9% of the patients were missed having PD pathology when only assessing the hands.

**Table 2:** Percentages of patients with PD activity at 6 and 12 months

	6 Months (*n* = 184)	12 Months (*n* = 152)
No joints with PD ≥ 1/≥ 2	16.5/44.0	21.4/50.9
PD ≥ 1/≥ 2 in hands as well as in other regions	40.1/18.7	37.1/14.5
PD ≥ 1/≥ 2 in hands but not in other regions	36.8/28.6	37.7/29.6
No PD ≥ 1/≥ 2 in hands but PD ≥ 1/≥ 2 in other regions	6.6/8.8	3.8/5.0

*Abbreviations: PD ≥ 1/≥ 2* Power Doppler score ≥ 1/≥ 2 in the wrist (radio-carpal, mid-carpal and radio-ulnar joints), *ECU* Extensor carpi ulnaris tendon, *MCP* Metacarpophalangeal joint, *PIP* Proximal interphalangeal joint, *MTP* Metatarsophalangeal joint, *TP* Tibialis posterior tendon, *n* Number of patients

PD activity is based on scores ≥ 1 or ≥ 2 detected in at least one joint/tendon in the hands (wrist, MCP joints 1–5, PIP joints 2–3, ECU tendon bilaterally) versus other regions (elbow, knee, ankle, MTP joints 1–5, TP bilaterally)

### Presence of US pathology in patients in remission with different composite scores

At 6 and 12 months, respectively, remission was found for DAS28 in 74 (40.7%)/59 (38.8%) patients, for CDAI in 37 (20.3%)/38 (25.0%) patients, for SDAI in 42 (23.1%)/42 (27.6%) patients and for Boolean in 38 (20.9%)/35 (23.0%) patients. Table 3 shows the percentages of patients in remission (based on composite scores) still having US pathology. Independent of type of composite score applied in the cohort, GS and PD pathology was present in a high proportion of patients.

**Table 3:** Patients in remission based on composite scores at 6- and 12-month follow-up

Remission	Hands (%)			Other regions (%)		
	GS ≥ 2	PD ≥ 1	PD ≥ 2	GS ≥ 2	PD ≥ 1	PD ≥ 2
6 months						
DAS28 (*n* = 74)	73.0	64.9	32.4	64.9	41.9	20.3
CDAI (*n* = 37)	62.2	62.2	27.0	62.2	35.1	16.2
SDAI (*n* = 42)	61.9	64.3	26.2	66.7	35.7	19.0
Boolean (*n* = 38)	68.4	63.2	26.3	71.1	31.6	13.2
12 months						
DAS28 (*n* = 59)	61.0	62.7	28.8	64.4	39.0	15.3
CDAI (*n* = 38)	47.4	57.9	15.8	50.0	34.2	13.2
SDAI (*n* = 42)	50.0	59.5	16.7	52.4	31.0	11.9
Boolean (*n* = 35)	42.9	54.3	17.1	54.3	31.4	14.3

*Abbreviations: GS ≥ 2* Greyscale score ≥ 2, *PD ≥ 1* Power Doppler score ≥ 1, *PD ≥ 2* Power Doppler score ≥ 2, *DAS28* Disease Activity Score based on 28 joints), *CDAI* Clinical Disease Activity Index, *SDAI* Simplified Disease Activity Index

Data represent percentages of patients still having ultrasound pathology in at least one joint/tendon in the hands (wrist [radio-carpal, mid-carpal, radio-ulnar], metacarpophalangeal joints 1–5, proximal interphalangeal joints 2–3, extensor carpi ulnaris tendon bilaterally) versus other regions (elbow, knee, ankle, metatarsophalangeal joints 1–5, tibialis posterior tendon bilaterally)

Of patients in remission based on composite score, ≥ 90.5% had PD activity (≥1 or ≥ 2) detected by assessing only the hands bilaterally (wrist [radio-carpal, mid-carpal, radio-ulnar joints], MCP joints 1–5, PIP joints 2–3 and ECU tendon) at 6- and 12-month follow-up (Table 4).

**Table 4:** Patients in composite score remission at 6 and 12 months with power Doppler activity in at least 1 joint/tendon of 36 joints/4 tendons

	6 months			12 months		
Remission	No. of patients	PD ≥ 1 (%)	PD ≥ 2 (%)	No. of patients	PD ≥ 1 (%)	PD ≥ 2 (%)
DAS28	74	91.9	91.9	59	94.9	96.6
CDAI	37	94.6	91.9	38	97.4	94.7
SDAI	42	92.9	90.5	42	97.6	95.2
Boolean	38	94.7	94.7	35	97.1	94.3

*Abbreviations: DAS28* Disease Activity Score based on 28 joints with erythrocyte sedimentation rate, *SDAI* Simplified Disease Activity Index, *CDAI* Clinical Disease Activity Index, *PD* Power Doppler

Percentage of patients where ultrasound assessments performed only of the hands (wrist (radiocarpal, midcarpal, radioulnar), metacarpophalangeal 1-5, proximal interphalangeal 2-3, extensor carpi ulnaris tendon bilaterally) would capture PD activity (≥1 or ≥ 2)

## Discussion

To improve the implementation of US as a clinical tool for evaluating inflammation, several reduced joint counts have been proposed to increase feasibility. They all include as a minimum the hands and feet [10–12]. The results of the present study support these findings because both regions appeared to be the most frequently inflamed joint regions, despite clinical remission. However, as reflected in the DAS28 assessment of clinical disease activity, the inclusion of the feet may prove difficult in a busy clinic. The objective of the present study was therefore to explore whether US examination of only the hands could be sufficient for detecting representative subclinical signs of inflammatory activity at the patient level. We found that > 90% of the patients with subclinical inflammation were identified by the presence of PD activity in the hands.

It has previously been established that US is more sensitive than clinical examination for the detection of subclinical disease, both at the time of diagnosis and in states of remission [28, 29]. The present study supports the existing literature by showing that, in patients fulfilling different clinical remission criteria, independent of the type of composite score applied, a high percentage still have US pathology at both 6 and 12 months [15, 18, 28]. Establishing subclinical inflammation has been shown to be important because US-detected inflammation (especially Doppler activity) in patients with RA during remission predicted erosive progression over time and was the best predictor of flare [15–17]. In addition, it has been demonstrated that the presence of Doppler activity is the best predictor for unsuccessful tapering of biologic treatment [30]. The importance of maintaining remission and avoiding flare is underlined by the increased likelihood of radiographic progression seen in patients with short-term remission compared with those achieving long-term remission. Furthermore, flare has also been shown to be associated with increased functional disability, pain and morning stiffness over time [31, 32].

In the present study, we chose a GS score ≥ 2 to define synovial hypertrophy. This cut-off was chosen because a GS score of 1 has been found to be a frequent finding in healthy persons [9, 33, 34] and appears less responsive to treatment in patients with RA [33]. In our cohort, US examination of only the hands captured the majority of patient with a GS score ≥ 2, and most of the pathology not detected was found in the MTP joints. Because the clinical experience is that GS score ≥ 2 is a frequent finding in MTP joints during follow-up of patients with RA, and because this degree of GS pathology also was found in MTP joints in healthy persons [9], the present study was not focused on the GS pathology in patients in clinical remission.

Two different cut-offs for PD activity were explored in the present study (PD ≥ 1 and PD ≥ 2) because both cut-offs have been shown to predict relapse or erosive progression [15, 30, 35]. However, we found the percentage of patients with the two cut-offs to be almost similar for the identification of patients with subclinical inflammation at both 6 and 12 months.

There is no consensus on the inclusion of tendons in US assessments of patients with RA. However, the ECU tendon was included in reduced joint scores [12], and in the present study this tendon was found to have PD activity more frequently than the large joints during follow-up. This supports a previous study where PD tenosynovitis in the hands was associated with shorter remission duration [20].

The present study was focussed on how to make US more feasible in daily clinical practise for detecting inflammation in patients in composite score remission. This topic was previously explored by Naredo et al. [36], who found GS and PD pathology in a high percentage of patients in remission by examining 44 joints, and they suggested assessing wrist, MCP, ankle and MTP joints to detect subclinical inflammation. In a recent study [37], 38 joints were assessed for subclinical inflammation, and PD ≥ 2 was found in at least one joint in 60% of patients in clinical remission. By reducing assessment to six joints (bilateral wrists and MCP joints 2–3), the researchers identified 75% of the patients with PD ≥ 2 [37], thus supporting our present findings based on assessing only the hands to explore subclinical inflammation. The six joints were explored post hoc in our present cohort, and PD ≥ 1 was found in 89% of all patients at 6 and 12 months, and PD ≥ 2 was found in 77% at 6 months and in 71% at 12 months. Among patients in remission (depending on the different composite scores), PD ≥ 1 was found in 55–60%/40–53% and PD ≥ 2 was found in 18–23%/5–17% at 6/12 months, respectively, which were lower than found by use of the present, more extended hand examination (Table 3).

In a previous study exploring patients with RA in clinical remission for the presence of US inflammation in the MCP joints, more than half of the joints had GS synovitis, and almost one-third had PD activity [38]. An important additional result was the presence of PD activity in half of the joints with US-detected erosion, supporting the association between US bone erosion and the persistence of subclinical inflammation. This finding strengthens our suggestion of performing US examination of the finger joints in patients with RA in clinical remission. However, researchers in future studies should continue to explore the optimal joints for a feasible US assessment to detect subclinical inflammation.

The limitations of the present study are the inclusion of only patients with established RA, as well as only patients initiating bDMARDs. Thus, whether the results are also representative of patients with recent-onset RA and receiving synthetic DMARD treatment should be explored in further studies. In addition, this was a single-centre study, which may limit the generalisability of the US findings. However, a standard US scoring system was used, and the experienced sonographer had shown high reliability for US scoring [25].

## Conclusions

The present study shows that, in patients with established RA on bDMARD treatment and in composite score clinical remission, US examination of only the hands is sufficient to detect > 90% of patients with subclinical inflammation. This suggests that US of the hands is a relevant clinical tool for this patient group during follow-up.

## Abbreviations

ACR: American College of Rheumatology
bDMARD: Biologic disease-modifying anti-rheumatic drug
CDAI: Clinical Disease Activity Index
CRP: C-reactive protein
DAS28(ESR): Disease Activity Score based on 28 joints with erythrocyte sedimentation rate
DMARD: Disease-modifying anti-rheumatic drug
ECU: Extensor carpi ulnaris
ESR: Erythrocyte sedimentation rate
GS: Greyscale
MC: Mid-carpal joint
MCP: Metacarpophalangeal
MTP: Metatarsophalangeal
PD: Power Doppler
PIP: Proximal interphalangeal
RA: Rheumatoid arthritis
RC: Radio-carpal
RU: Radio-ulnar
SDAI: Simplified Disease Activity Index
T2T: Treat-to-target strategy
TP: Tibialis posterior
US: Ultrasound
VAS: Visual analogue scale
